# Characterizing the binding of TC-5619 and encenicline on the alpha7 nicotinic acetylcholine receptor using PET imaging in the pig

**DOI:** 10.3389/fnimg.2024.1358221

**Published:** 2024-03-27

**Authors:** Janus H. Magnussen, Anders Ettrup, Szabolcs Lehel, Dan Peters, Agnete Dyssegaard, Morten S. Thomsen, Jens D. Mikkelsen, Gitte M. Knudsen

**Affiliations:** ^1^Neurobiology Research Unit, Rigshospitalet, Copenhagen, Denmark; ^2^Faculty of Health and Medical Sciences, University of Copenhagen, Copenhagen, Denmark; ^3^PET and Cyclotron Unit, Rigshospitalet, Copenhagen, Denmark; ^4^DanPET AB, Malmö, Sweden; ^5^Department of Drug Design and Pharmacology, Faculty of Health and Medical Sciences, University of Copenhagen, Copenhagen, Denmark; ^6^Institute of Neuroscience, University of Copenhagen, Copenhagen, Denmark; ^7^Department of Clinical Medicine, University of Copenhagen, Copenhagen, Denmark

**Keywords:** positron emission tomography (PET), alpha7, nicotinic acetylcholine receptors, autoradiography, occupancy study, cognitive impairment

## Abstract

The alpha7 nicotinic acetylcholine receptor (α7-nAChR) has has long been considered a promising therapeutic target for addressing cognitive impairments associated with a spectrum of neurological and psychiatric disorders, including Alzheimer's disease and schizophrenia. However, despite this potential, clinical trials employing α7-nAChR (partial) agonists such as TC-5619 and encenicline (EVP-6124) have fallen short in demonstrating sufficient efficacy. We here investigate the target engagement of TC-5619 and encenicline in the pig brain by use of the α7-nAChR radioligand ^11^C-NS14492 to characterize binding both with *in vitro* autoradiography and *in vivo* occupancy using positron emission tomography (PET). *In vitro* autoradiography demonstrates significant concentration-dependent binding of ^11^C-NS14492, and both TC-5619 and encenicline can block this binding. Of particular significance, our *in vivo* investigations demonstrate that TC-5619 achieves substantial α7-nAChR occupancy, effectively blocking approximately 40% of α7-nAChR binding, whereas encenicline exhibits more limited α7-nAChR occupancy. This study underscores the importance of preclinical PET imaging and target engagement analysis in informing clinical trial strategies, including dosing decisions.

## Introduction

**Figure 1:**
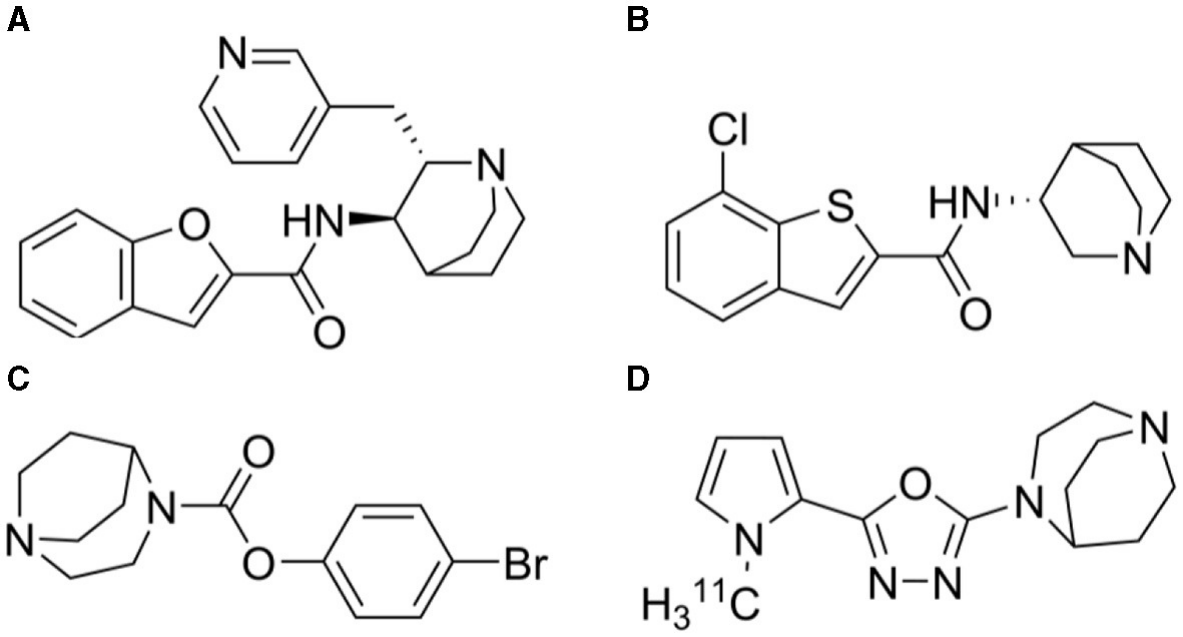
Chemical structures of **(A)** TC-5619 (*N*-((2*S*,3*R*)-2-(pyridin-3-ylmethyl)quinuclidin-3-yl)benzofuran-2-carboxamide) **(B)** Encenicline [(*R*)-7-chloro-*N*-(quinuclidin-3-yl)benzo[*b*]thiophene-2-carboxamide] **(C)** SSR180711 (4-bromophenyl 1,4-diazabicyclo[3.2.2]nonane-4-carboxylate) and **(D)** ^11^C-NS14492 [2-(1,4-diazabicyclo[3.2.2]nonan-4-yl)-5-(1-(methyl-^11^C)-1*H*-pyrrol-2-yl)-1,3,4-oxadiazole].

The alpha7 nicotinic acetylcholine receptor (α7-nAChR) is a homopentameric ligand-gated ion channel that is involved in the regulation of cognitive processes in normal conditions as well as is in the pathophysiology of some brain disorders. The receptor is composed of five identical α7 subunits, yielding an equal number of binding sites (Dani and Bertrand, 2007; Li et al., 2011). This receptor is widely distributed in the central nervous systems (CNS) and found with high densities in regions associated with cognitive functions (Tribollet et al., 2004; Wessler and Kirkpatrick, 2008). Of note, there is substantial evidence for the presence of heteromeric α7-nAChRs in mammalian CNS, where α7 subunits co-assemble with β2 subunits to form functional α7β2-nAChR (Wu et al., 2016). The exact implications of the existence of this receptor are unknown. The homopentameric α7-nAChR has been investigated as a potential therapeutic target for addressing cognitive impairments associated with neurological and psychiatric diseases, including Alzheimer's disease and schizophrenia (Wallace and Porter, 2011). Over the past 20 years, several compounds have been developed to selectively target the α7-nAChR, demonstrating promising effects in enhancing cognitive functions in animal models (Thomsen et al., 2010). However, a significant translational challenge persists, as these encouraging preclinical outcomes have not translated into corresponding benefits in human clinical trials (Lewis et al., 2017). Some notable examples are TC-5619 from Targacept (Figure 1A), encenicline (EVP-6124) from Forum Pharmaceuticals (Figure 1B), and SSR180711 from Sanofi (Figure 1C). *In vitro*, TC-5619 is a full and potent α7-nAChR agonist reported to be effective in rodent models of schizophrenia (Hauser et al., 2009). Initially, in an exploratory 12-week randomized clinical phase 2 trial involving 185 subjects with schizophrenia, TC-5619 showed promising effects on cognitive endpoints and negative symptoms compared to placebo (Lieberman et al., 2013). However, in a clinical phase 2 trial lasting 24 weeks, TC-5619 failed to meet the primary outcome measure of change from baseline on the Scale for the Assessment of Negative Symptoms (SANS) compared to placebo (Walling et al., 2016). Additionally, TC-5619 did not demonstrate improvement in the key secondary measures of cognitive function (ClinicalTrials.gov identifier: NCT01488929) and further development was stopped (Targacept, 2013). SSR180711 is a partial α7-nAChR agonist (Biton et al., 2007) that has been shown to improve long-term and short-term episodic memory and spatial working memory in rodents (Pichat et al., 2007). The compound was tested in a placebo-controlled phase 2 clinical trial, spanning 4 weeks and three different dosage regimens, in patients with mild Alzheimer's disease (NCT00602680). However, the trial was prematurely terminated in 2008 due to an inadequate risk-benefit ratio, as documented on clinicaltrials.gov. Encenicline is a partial α7-nAChR agonist *in vitro* and it reverses a scopolamine-induced memory deficit *in vivo* in rats (Prickaerts et al., 2012). Despite encenicline showing effects in a proof-of-concept, randomized trial in patients with schizophrenia (Preskorn et al., 2014) and in mild-to-moderate Alzheimer's disease patients on functional and cognitive skills compared to placebo (Deardorff et al., 2015), this outcome was not confirmed in two larger global clinical phase 3 trials as the trials were put on hold due to severe gastrointestinal adverse effects (Alzforum, 2016). Further, encenicline is so far the only compound targeting the α7-nAChR that has been evaluated in a large-scale clinical phase 3 trial in schizophrenia patients, but no effect could be seen (Brannon, 2019) [see review by Terry and Callahan (2020)]. The lack of success in clinical trials involving α7-nAChR ligands in Alzheimer's disease or schizophrenia has reduced the enthusiasm for this target and consequently, many pharmaceutical companies have discontinued their research efforts in this field (Bertrand and Terry, 2018).

However, before discarding α7-nAChR as a viable target, it is worth noting that despite several large clinical trials, little information regarding the compounds' blood-brain barrier permeability, target involvement and the level of α7-nAChR occupancy has been published. For these purposes, molecular brain imaging emerges as a powerful tool capable of providing invaluable insights into target engagement and occupancy. Several α7-nAChR positron emission tomography (PET) radioligands have so far been tested: ^18^F-ASEM was developed as an α7-nAChR antagonist with suitable binding properties (Horti et al., 2014) and tested in 21 healthy non-smoking volunteers and in 6 males with schizophrenia (Wong et al., 2014, 2018). We have also validated ^11^C-NS14492 (Figure 1D) as a selective α7-nAChR agonist PET radioligand capable of measuring α7-nAChR occupancy of SSR180711 and unlabelled NS14492 in the pig brain (Ettrup et al., 2011b). This compound has been further described and validated as a tritiated *in vitro* radioligand (Magnussen et al., 2015). In our study, we chose pigs as experimental animal due to their physiological and anatomical similarities to humans, facilitating translational relevance. Furthermore, the presence of the nAChR in the pig brain has been well-documented, particularly validated through numerous PET experiments, including a recent study employing ^18^F-ASEM, confirming the comparability of nAChR expression patterns between humans and pigs (Donat et al., 2020). Here, we report the effect of TC-5619 and encenicline on ^11^C-NS14492 binding in the pig brain using both *in vitro* autoradiography and *in vivo* PET imaging to elucidate the target occupancy for these molecules.

## Methods

### Compounds

^11^C-NS14492 was produced as described previously (Ettrup et al., 2011b). Briefly, the radioligand was produced by transferring ^11^C-methyl triflate in a stream of helium to a vial containing desmethyl-NS14492 fumarate dissolved in 300 μL of acetone and 10 μL of 1 M tetrabutylammonium hydroxide in methanol before being heated to 60°C for 3 min prior to high-performance liquid chromatography purification. The specific radioactivity of the radioligand was in the range of 84–152 GBq/μmol, calculated at the end of synthesis, and the radiochemical purity was >99% (*n* = 8). Encenicline, TC-5619, SSR180711, desmethyl-NS14492, and NS14492 were synthesized at NeuroSearch A/S, (Ballerup, Denmark). All other reagents were purchased from Sigma-Aldrich (Brøndby, Denmark) and were of analytical grade.

### Animal procedures

All animal procedures were approved by the Danish Council for Animal Ethics (journal no. 2012-15-2934-00156). For this study, four female Danish Landrace pigs (*Sus scrofa*) (mean weight ± SD, 19 ± 3 kg) were used. The animals were sourced from a local farm, placed in standard housing conditions, and given a minimum acclimatization period of 1 week in the veterinary facilities. At the day of the experiment, but before scanning, the pigs were treated with midazolam [0.5 mg/kg intramuscular (i.m.)] and anesthesia was subsequently induced with an i.m. injection of 1 mL/kg Zoletil veterinary mixture [6.25 Pt. xylazine (20 mg/mL) + 1.25 Pt. ketamine (100 mg/mL) + 2 Pt. butorphanol (10 mg/mL) + 2 Pt. methadone (10 mg/mL); Virbac, Kolding, Denmark]. Hereafter, anesthesia was maintained with a constant propofol infusion [10 mg/kg/h intravenous (i.v.); B. Braun, Melsungen, Germany]. During anesthesia, animals were endotracheally intubated and ventilated. Venous access was granted through the peripheral milk veins, and an arterial line for blood sampling was inserted in the femoral artery after a minor incision. Vital parameters (heart rate, body temperature, blood pressure, oxygen saturation, and end-tidal pCO_2_) were continuously monitored during the scans. The pigs were euthanized immediately after scanning with an i.v. injection of pentobarbital.

### *In vitro* autoradiography

*In vitro* autoradiography was performed on post-mortem brain tissue from pigs. Coronal 12 μm sections of pig frontal cortex and thalamus/parietal cortex were cut on a HM500OM Cryostat (Microm Intl GmbH, Walldorf, Germany) at −20°C, thaw-mounted on SuperFrost Plus glass slides (Thermo Scientific, Hvidovre, Denmark), air-dried, and stored at −80°C until use. Autoradiography was conducted at room temperature with 10 nM ^11^C-NS14492 for total binding, and non-specific binding was determined in the presence of either TC-5619 or encenicline or SSR180711 (10 μM) used in pre-incubation buffer (50 mM tris–HCl, 4 mM CaCl_2_, 0.1% bovine serum albumin (BSA), 120 mM NaCl, 5 mM KCl, pH 7.4). Sections were then incubated for 30 min in 50 mM tris-HCl buffer (pH 7.4) containing 120 mM NaCl, 5 mM KCl, 1 mM MgCl_2_, 2.5 mM CaCl_2_, 0.1% BSA, and 10 nM ^11^C-NS14492 and washed 2 × 2 min in ice-cold buffer followed by 20 seconds in ice-cold distilled water. Sections were exposed to an imaging plate (IP) (Fujifilm, Tokyo, Japan) in a BAS-2040 cassette overnight. The IP was scanned in a BAS-2500 (Fujifilm) image reader. Image analysis was done with ImageJ analysis software (http://rsb.info.nih.gov/ij/).

### *In vivo* PET imaging

PET experiments were performed with a high-resolution research tomography (HRRT) scanner (Siemens Medical Solutions, Munich, Germany) as previously described (Ettrup et al., 2011b). Briefly, ^11^C-NS14492 was given as an i.v. bolus injection (injected dose, 458 ± 93 MBq; injected cold mass, 0.8 ± 0.4 μg, *n* = 8), and the pigs were scanned at baseline for 90 min in list mode. After a baseline scan, the animals were given a bolus injection of either TC-5619 (3 mg/kg i.v., *n* = 2) or encenicline (3 mg/kg i.v., *n* = 2) dissolved in 10 mL saline and rescanned 30 min later using the same PET protocol. Whole blood radioactivity was continuously measured for the first 30 min after radioligand injection using an ABSS autosampler (Allogg Technology, Strängnäs, Sweden). Additionally, manual sampling of arterial whole blood (8–13 mL) occurred at intervals of 2.5, 5, 10, 20, 30, 50, 70, and 90 min after injection. Subsequently, radioactivity levels in both whole blood and plasma were quantified using a Cobra 5,003 well counter (Packard Instruments).

The PET data was reconstructed as previously reported (Ettrup et al., 2011a), and the individual summed images of all counts during the 90-min scan time were co-registered to a standardized MRI atlas of the Danish Landrace pig brain using the software Register, as previously described (Kornum et al., 2009). Radioactive concentrations (Bq/mL) were extracted from specific brain regions of both hemispheres [cerebellum, cortex, hippocampus, thalamus (average of lateral and medial), and striatum (average of caudate and putamen)]. Time-activity curves of radioactive concentrations in volumes of interest were normalized to the injected dose and adjusted for body weight. ^11^C-NS14492 metabolism was measured with HPLC analysis with online radioactivity detection, as previously described (Gillings, 2009). For kinetic modeling purposes, an average metabolite curve for all 8 scans was generated and used to correct plasma activity in the individual scans for the parent compound fraction, thereby obtaining the ^11^C-NS14492 arterial input function. Volumes of distribution (*V*_T_) for selected regions were calculated using PMOD software (version 3.0; PMOD Technologies Inc.) applying a Logan graphical analysis with arterial input function (Logan et al., 1990). Because there is no suitable brain reference region devoid of α7-nAChRs, occupancy was measured using the revisited Lassen plot (Cunningham et al., 2010). In the four blocking scans, occupancies of the receptor by encenicline or TC-5619 were calculated as the slope of the occupancy plot visualizing *V*_T_(baseline) – *V*_T_(blocking) as a linear function of *V*_T_(baseline) in the specific brain regions (cortex, thalamus, striatum, cerebellum, and hippocampus). The non-displaceable distribution volume (*V*_ND_) was determined by the x-intercept of the regression line.

### Statistical analyses

All statistical tests were performed using GraphPad Prism version 6.0 (GraphPad Software, San Diego, USA). *P*-values below 0.05 were considered statistically significant. Results are presented as mean ± standard error of the mean (SEM) unless stated otherwise.

## Results

### Regional binding and blocking effects of TC-5619, encenicline, and SSR180711 in cortical pig sections

**Figure 2:**
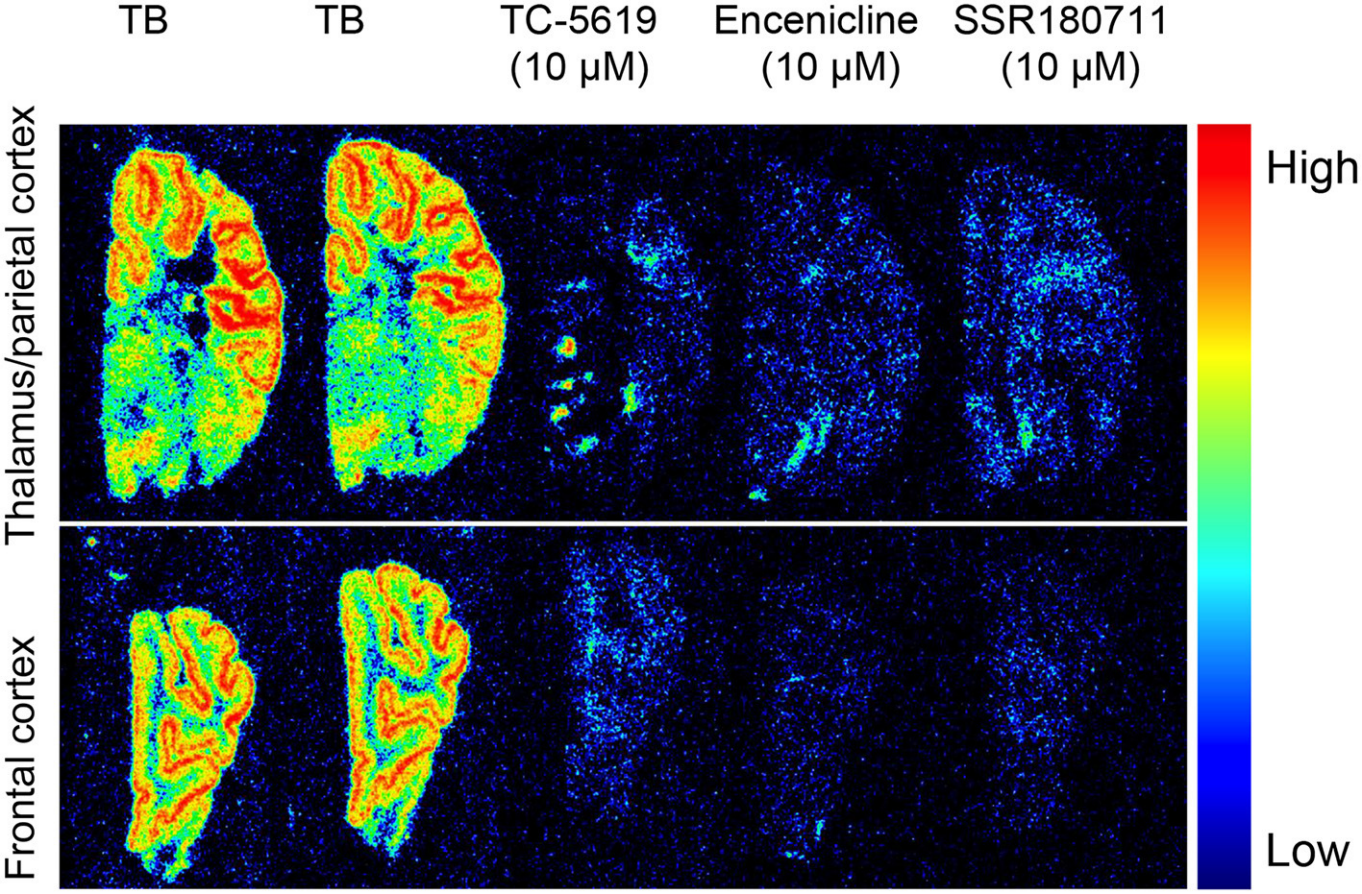
*In vitro* autoradiography with ^11^C-NS14492 in 12 μm coronal pig sections containing thalamus **(upper panel)** and frontal cortex **(lower panel)**. Total binding (TB) was measured with 10 nM ^11^C-NS14492. Non-specific binding shown with TC-5619 (10 μM), Encenicline (10 μM) and SSR180711 (10 μM).

To investigate the binding properties of TC-5619, encenicline and SSR180711 we performed *in vitro* autoradiography in coronal pig sections using 10 nM ^11^C-NS14492 (Figure 2). We observed laminar binding in cortical layers and less binding in white matter. Further, we observed an almost complete blocking with 10 μM of all three compounds: TC-5619, encenicline, and SSR180711.

### Imaging *in vivo* binding profiles and blocking effects of TC-5619 and encenicline

**Figure 3:**
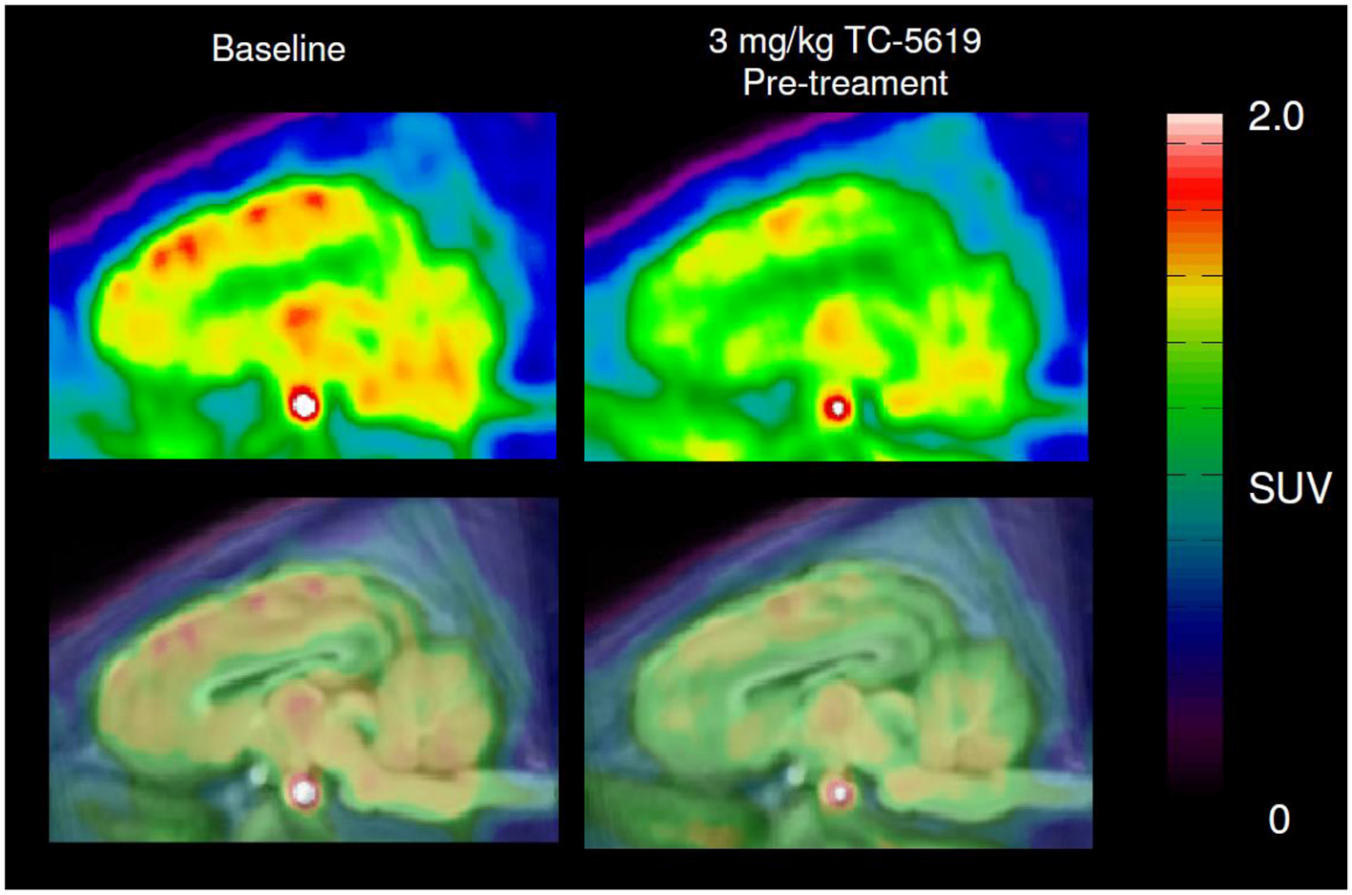
Representative sagittal PET images of ^11^C-NS14492 PET scans before and after TC-5619 pre-treatment. Summed and averaged over 0–90 min PET image **(top panel)** overlaid MRI-based pig brain atlas **(bottom panel)**. SUV; standardized uptake value.

**Figure 4:**
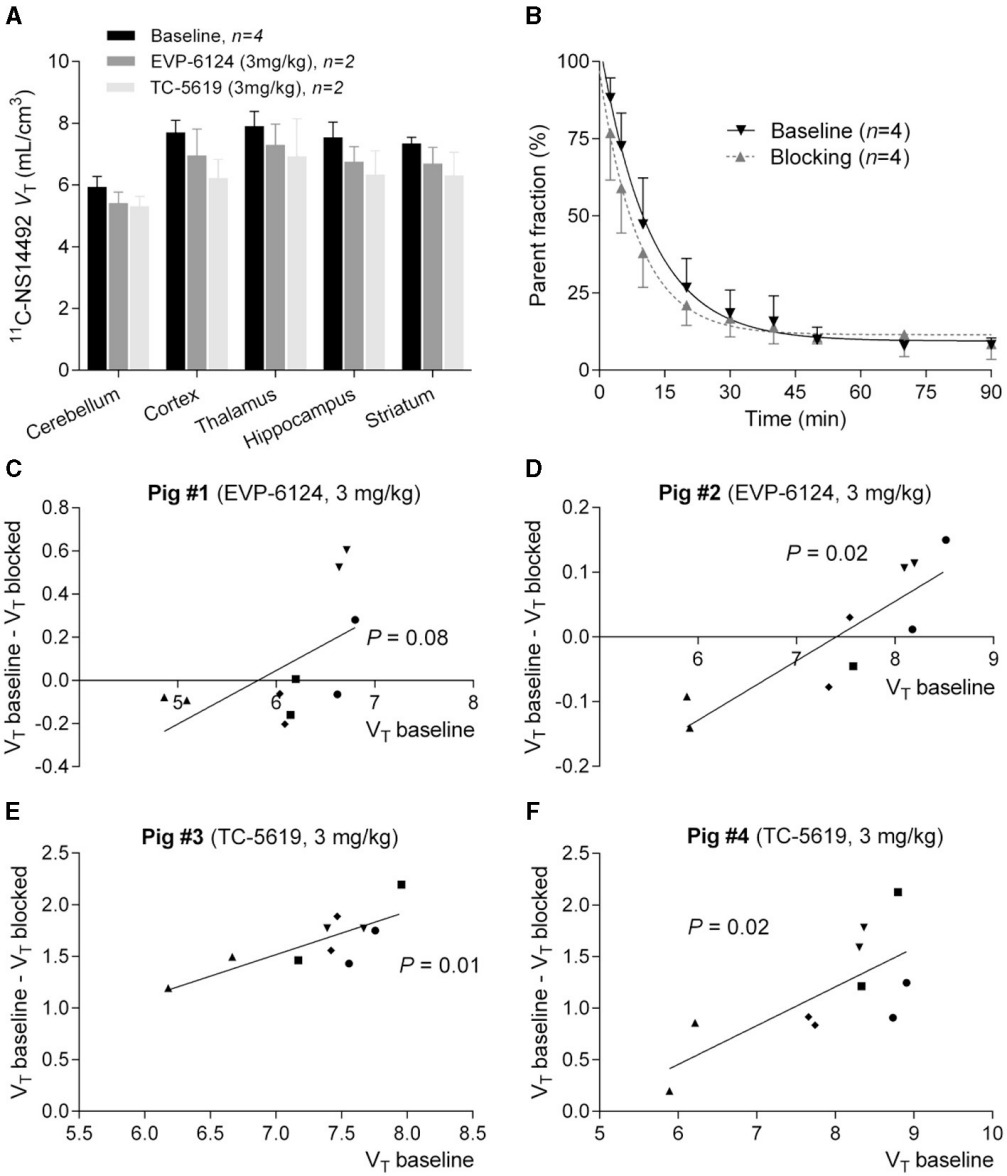
**(A)** Logan plot *V*_T_'s of ^11^C-NS14492 in five brain regions are shown at baseline and after intervention with EVP-6124 (Encenicline) (3 mg/kg i.v. administered 30 minutes before second scan) or TC-5619 (3 mg/kg i.v. administered 30 min before second scan). Bars indicate mean ± SEM. **(B)** Relative radioactive parent compound in pig plasma as a function of time after i.v. injection of ^11^C-NS14492. Average of measurements from 8 PET scans is shown. Baseline scans (black, *n* = 4) and challenge scans (gray, *n* = 4) with 3 mg/kg Encenicline (*n* = 2) or 3 mg/kg TC-5619 (*n* = 2) is shown. Solid lines (black and gray) correspond to a single exponential decay function fitted to the data. **(C–F)** Occupancy plots of ^11^C-NS14492 regional left and right *V*_T_'s at baseline and in intervention scan for individual pigs. Receptor occupancy by EVP-6124 (Encenicline) **(C, D)** or TC-5619 **(E, F)** is measured as slope of regression line. ^11^C-NS14492 *V*_ND_ is found as x-axis intercept. Statistical test results (*P-*values) for slope not equal to zero is shown for the individual regression lines. ▾ = cortex; • = thalamus; ▴ = cerebellum; ■ = hippocampus; ♦ = striatum.

After a bolus injection of ^11^C-NS14492, we observed heterogeneous brain uptake of radioactivity (Figure 3) with the highest uptake in thalamus and cortical areas, intermediate uptake in the striatum and the lowest uptake in the cerebellum. Pre-treatment with 3 mg/kg TC-5619 or 3 mg/kg encenicline was associated with a lower *V*_T_ in all measured regions (Figure 4A). Whereas pre-treatment with 3 mg/kg TC-5619 resulted in an occupancy of 38%−42% (Figures 4C, D), pre-treatment with 3 mg/kg encenicline resulted in less than in average 10% occupancy. In one pig, the slope of the linear regression was not significantly different from zero (Figures 4E, F). The average *V*_ND_ was 5.3 ± 1.7 mL/cm^3^ (*n* = 3).

After i.v. injection, the parent fraction of ^11^C-NS14492 declined rapidly, and after 7 min, approximately 50% of radioactivity in plasma was attributable to parent ^11^C-NS14492 (Figure 4B). We detected no radiolabelled lipophilic metabolites of ^11^C-NS14492 in the plasma, as indicated by lack of distinct peaks in the lipophilic range on the radiochromatograms (data not shown).

## Discussion and conclusion

Here, we present *in vitro* receptor autoradiography and *in vivo* ^11^C-NS14492 PET data on α7-nAChR ligands in the pig brain. The *in vitro* autoradiography revealed laminar binding in cortical layers with a clear discrimination between gray and white matter binding, consistent with previous studies (Gotti et al., 2006). The three α7-nAChR ligands TC-5619, encenicline, and SSR180711 were all able to block ^11^C-NS14492 binding *in vitro*, supporting their effectiveness as competitive ligands for the α7-nAChR orthosteric site.

With *in vivo* PET imaging we find that 3 mg/kg TC-5619 given i.v. results in 40% occupancy at the α7-nAChR whereas the same dose of encenicline results in negligible occupancy. By using an *in vitro* homogenate binding assay with ^3^H-NS14492, we previously found *K*_i_ values for TC-5619 and encenicline of 0.063 nM and 0.194 nM respectively (Magnussen et al., 2015). That is, given the observed occupancy of TC-5619, one could expect to find that a dose of 3 mg/kg encenicline would result in 16% α7-nAChR occupancy.

Several factors could explain the low *in vivo* occupancy of encenicline in the pig brain. If encenicline displayed a very rapid drug clearance it may have insufficient time to be taken up by the brain. We know from rats that encenicline has good brain penetration after oral administration (0.3 mg/kg), with brain-to-plasma ratios of approximately 2 between 1 and 4 h and 5 at 8 h (Prickaerts et al., 2012), indicating that the plasma clearance of encenicline is considerably faster than the brain uptake and receptor equilibration. In humans, and after oral administration, encenicline has a long plasma half-life of 50–65 h (Barbier et al., 2015), but pharmacokinetic differences between humans and pigs could be a factor as well as differences between oral and intravenous administration. Another explanation could lie with the known interspecies differences in blood-brain barrier permeability for compounds like encenicline and TC-5619 among rats, pigs, and potentially humans (Syvanen et al., 2009; Deo et al., 2013; Stanimirovic et al., 2015). This theory could partially explain the reason for encenicline's clinical trial failure. Furthermore, an extensive review of the existing literature and public domain resources has revealed no documented evidence demonstrating the ability of encenicline to cross the blood-brain barrier in humans.

Our investigation in the pig model reveals that TC-5619 effectively penetrates the blood-brain barrier and exhibits significant binding to the α7-nAChR with a reasonable occupancy. However, it is noteworthy that no prior studies have definitively established an optimal range of α7-nAChR occupancy necessary for eliciting therapeutic effects. This lack of established benchmarks raises the possibility that the failure of TC-5619 to produce significant pro-cognitive effects in clinical trials may stem from the selection of suboptimal dosages. Interestingly, previous research on selective α7-nAChR agonists, including encenicline (Keefe et al., 2015), TC-5619 (Hauser et al., 2009), as well as other compounds such as AZD0328 (Castner et al., 2011), DMXB-A (Olincy et al., 2006), and PHA543613 (Yang et al., 2013) has indicated a trend where the pro-cognitive effects peak at lower doses, following an inverted U-shaped dose-response curve. This phenomenon suggests that while lower doses may elicit optimal cognitive enhancement, escalating doses beyond this threshold could lead to diminishing effect or even receptor desensitization, thereby limiting further cognitive improvement. The implications of this dose-response pattern are profound, particularly in the context of human clinical trials. The absence of a clear understanding of optimal dosing presents a considerable challenge, underscoring the need for robust pre-clinical tools for dose finding.

Our study is not without limitations. First, it was not possible to measure drug concentrations during the PET studies so an eventual ultra-fast drug metabolism of encenicline cannot be excluded. As discussed above, this could potentially result in an underestimation of its occupancy. Secondly, only 4 animals were used in total limiting the statistical power of the study. However, each animal served as its own control, and we measured five distinct occupancy regions in each animal. Thirdly, we administered both TC-5619 and encenicline at 3 mg/kg but cannot exclude that an even higher dose of encenicline could have returned a larger occupancy.

In conclusion, we find that the two α7-nAChR ligands TC-5619 and encenicline, when given at equal doses, display different α7-nAChR occupancy *in vivo* in the pig brain. Our findings underscore the significance of utilizing PET radiotracers in CNS pre-clinical drug development in assessing crucial factors such as blood-brain barrier permeability and target engagement, as well as in aiding dose finding for potential therapeutic agents. By proposing the establishment of target occupancy through PET experiments prior to embarking on clinical trials, we advocate for a more precise dosing strategy for emerging α7-nAChR selective drug candidates. This proactive approach has the potential to mitigate uncertainties surrounding dosing regimens, thereby enhancing the likelihood of therapeutic success while minimizing adverse effects.

## Data availability statement

The raw data supporting the conclusions of this article will be made available by the authors, without undue reservation.

## Ethics statement

The animal studies were approved by the Danish Council for Animal Ethics. The studies were conducted in accordance with the local legislation and institutional requirements. Written informed consent was obtained from the owners for the participation of their animals in this study.

## Author contributions

JHM: Conceptualization, Data curation, Formal analysis, Investigation, Methodology, Visualization, Writing – original draft, Writing – review & editing. AE: Formal analysis, Methodology, Writing – review & editing. SL: Formal analysis, Software, Writing – review & editing. DP: Writing – review & editing. AD: Data curation, Formal Analysis, Writing – review & editing. MT: Methodology, Writing – review & editing. JDM: Conceptualization, Data curation, Formal analysis, Funding acquisition, Methodology, Resources, Supervision, Writing – review & editing. GK: Conceptualization, Data curation, Formal analysis, Funding acquisition, Methodology, Resources, Supervision, Writing – review & editing.
